# Prediction of immunochemotherapy response for diffuse large B‐cell lymphoma using artificial intelligence digital pathology

**DOI:** 10.1002/2056-4538.12370

**Published:** 2024-04-08

**Authors:** Jeong Hoon Lee, Ga‐Young Song, Jonghyun Lee, Sae‐Ryung Kang, Kyoung Min Moon, Yoo‐Duk Choi, Jeanne Shen, Myung‐Giun Noh, Deok‐Hwan Yang

**Affiliations:** ^1^ Department of Radiology Stanford University School of Medicine Stanford CA USA; ^2^ Department of Hematology‐Oncology Chonnam National University Hwasun Hospital Hwasun Republic of Korea; ^3^ Department of Medical and Digital Engineering Hanyang University College of Engineering Seoul Republic of Korea; ^4^ Department of Nuclear Medicine Chonnam National University Hwasun Hospital and Medical School Hwasun‐gun Republic of Korea; ^5^ Division of Pulmonary and Allergy Medicine, Department of Internal Medicine Chung‐Ang University Hospital, Chung‐Ang University College of Medicine Seoul Republic of Korea; ^6^ Artificial Intelligence, Ziovision Co., Ltd. Chuncheon Republic of Korea; ^7^ Department of Pathology Chonnam National University Medical School Gwangju Republic of Korea; ^8^ Department of Pathology and Center for Artificial Intelligence in Medicine & Imaging Stanford University School of Medicine Stanford CA USA; ^9^ Department of Pathology School of Medicine, Ajou University Suwon Republic of Korea

**Keywords:** diffuse large B‐cell lymphoma (DLBCL), digital pathology, deep learning, multi‐modal prediction model

## Abstract

Diffuse large B‐cell lymphoma (DLBCL) is a heterogeneous and prevalent subtype of aggressive non‐Hodgkin lymphoma that poses diagnostic and prognostic challenges, particularly in predicting drug responsiveness. In this study, we used digital pathology and deep learning to predict responses to immunochemotherapy in patients with DLBCL. We retrospectively collected 251 slide images from 216 DLBCL patients treated with rituximab, cyclophosphamide, doxorubicin, vincristine, and prednisone (R‐CHOP), with their immunochemotherapy response labels. The digital pathology images were processed using contrastive learning for feature extraction. A multi‐modal prediction model was developed by integrating clinical data and pathology image features. Knowledge distillation was employed to mitigate overfitting on gigapixel histopathology images to create a model that predicts responses based solely on pathology images. Based on the importance derived from the attention mechanism of the model, we extracted histological features that were considered key textures associated with drug responsiveness. The multi‐modal prediction model achieved an impressive area under the ROC curve of 0.856, demonstrating significant associations with clinical variables such as Ann Arbor stage, International Prognostic Index, and bulky disease. Survival analyses indicated their effectiveness in predicting relapse‐free survival. External validation using TCGA datasets supported the model's ability to predict survival differences. Additionally, pathology‐based predictions show promise as independent prognostic indicators. Histopathological analysis identified centroblastic and immunoblastic features to be associated with treatment response, aligning with previous morphological classifications and highlighting the objectivity and reproducibility of artificial intelligence‐based diagnosis. This study introduces a novel approach that combines digital pathology and clinical data to predict the response to immunochemotherapy in patients with DLBCL. This model shows great promise as a diagnostic and prognostic tool for clinical management of DLBCL. Further research and genomic data integration hold the potential to enhance its impact on clinical practice, ultimately improving patient outcomes.

## Introduction

Diffuse large B‐cell lymphoma (DLBCL) is the most common subtype of aggressive non‐Hodgkin lymphoma. In total, 60% of patients could be cured with rituximab, cyclophosphamide, doxorubicin, vincristine, and prednisone (R‐CHOP); however, the remaining 40% of patients with chemorefractory disease eventually relapse and have a dismal prognosis [1]. DLBCL poses a unique diagnostic challenge due to its inherent clinical and pathological heterogeneity. This heterogeneity translates into variable clinical outcomes, emphasising the importance of precise diagnostic strategies.

Histopathological examination based on tissue morphology has been the bedrock of lymphoma diagnosis for decades. The characteristic diffuse growth pattern of large B cells effacing the lymph node architecture serves as a pivotal diagnostic hallmark. Although morphology provides essential clues, immunophenotyping (usually by immunohistochemistry) is vital for a definitive diagnosis. DLBCL cells typically express pan‐B cell markers, such as CD19, CD20, CD22, and CD79a. DLBCL can be further sub‐classified based on its cell of origin into germinal centre B‐cell‐like (GCB) and activated B‐cell‐like (ABC) using markers such as CD10, BCL6, and MUM1. This distinction has prognostic implications and can be used to guide treatment strategies. Some DLBCLs express other markers, such as BCL2, MYC, and CD30. Double or triple expression of BCL2, MYC, and BCL6 has diagnostic and prognostic implications. A *MYC* rearrangement concurrent with a rearrangement in *BCL2*, *BCL6*, or both occurs in 4–8% of DLBCL cases. These cases are referred to as double‐ or triple‐hit lymphomas, which are now classified as high‐grade B‐cell lymphomas with *MYC* and *BCL2* and/or *BCL6* rearrangements and are associated with poor clinical outcomes after R‐CHOP therapy [2, 3, 4].

DLBCL represents a highly heterogeneous diagnostic category in terms of morphology, genetics, and biological behaviour [5]. Several efforts have been made to clarify this heterogeneity and to predict the clinical outcomes of DLBCL. Standard prognostic factors for DLBCL include the International Prognostic Index (IPI), imaging with PET/CT, and FISH for *MYC* and *BCL2* rearrangement. The IPI model can be employed to identify five factors to predict survival: age >60, elevated serum lactate dehydrogenase (LDH), Eastern Cooperative Oncology Group performance status ≥2, Ann Arbor stage III or IV, and number of involved extranodal sites ≥2. Four risk groups were identified with predicted 5‐year survival rates of 73%, 51%, 43%, and 26%, respectively [6]. 18‐Fluorodeoxyglucose (^18^FDG)‐PET/CT is a highly sensitive method for detecting sites involved in DLBCL, and baseline staging and response assessment using PET/CT are significantly associated with survival [7, 8]. Recently, gene expression profiling has been investigated as a novel prognostic factor. Gene expression profiling can classify cases into two distinct subtypes, the GCB subtype and the ABC subtype, which are relevant because targeted agents could be active in one subtype [9, 10]. Subsequently, molecular classification based on targeted deep sequencing can further subdivide DLBCL into MCD, N1, A53, BN2, ST2, and EZB [11, 12]. Despite these advances in the genetic classification, the clinical heterogeneity of DLBCL has not yet been fully defined.

Traditional diagnostic methods largely depend on histopathological examination, which, while invaluable, has inherent challenges, such as inter‐observer variability and the time‐intensive nature of the evaluation. The advent of digital pathology, which entails scanning conventional glass slides to produce digital slides, has revolutionised the landscape of pathological diagnostics. This innovation not only facilitates remote consultations and integrated multidisciplinary team reviews but also paves the way for advanced computational analyses. Concurrently, deep learning, a subset of machine learning and artificial intelligence (AI), has burgeoned in various fields of medicine, showing exceptional success in image‐recognition tasks. Marrying the high‐resolution, data‐rich environment of digital pathology with the robust pattern recognition capabilities of deep learning models, particularly convolutional neural networks (CNNs), promises a transformative shift in haematopathology. Such synergy could potentially offer enhanced diagnostic accuracy, consistency, and speed, thereby addressing some of the limitations of conventional methods. Previous studies using digital pathology and AI reported promising results regarding disease diagnosis, sub‐classification, and outcome prediction in other solid cancers [13, 14, 15, 16, 17]. For DLBCL, several studies have utilised AI to improve the diagnostic accuracy and detection of *MYC* translocation [18, 19, 20, 21]. However, no study has demonstrated the prognostic implications of digital pathology using AI. Furthermore, there are no models that predict responsiveness to chemotherapy or studies using digital pathology to predict prognosis.

In this study, we investigated the histopathologic features that predict the response to immunochemotherapy in patients with DLBCL using digital pathology images at diagnosis with clinical data through deep multiple instance learning (MIL). This new prediction model, based on pathological findings, could provide a background for determining frontline treatment strategies for DLBCL.

## Methods

### Study population and histopathology dataset

**Figure 1:**
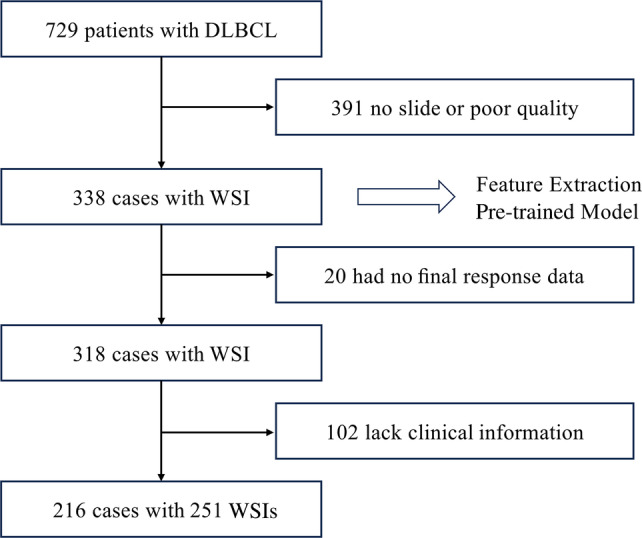
Flowchart of case selection with inclusion and exclusion criteria.

A total of 729 patients newly diagnosed with DLBCL and treated with R‐CHOP between 2005 and 2020 at a single institution (Chonnam National University Hwasun Hospital) were enrolled to develop and internally validate the DLBCL model. All patients received 3–8 cycles of R‐CHOP, and patients with initial bulky disease received consolidative involved‐field radiotherapy (IFRT) after immunochemotherapy. Clinical and pathological data, including age, performance, symptoms, LDH level, extranodal involvement, Ann Arbor stage, spleen and bone marrow involvement in baseline FDG‐PET/CT, IPI score, revised IPI, bulky disease, Bcl‐2, IFRT, interim and end‐of‐treatment PET/CT response, and total cycles of R‐CHOP, were collected from all available electronic medical records. The tissue slides from 729 patients were reviewed. As the slides before the year 2020 were old, the staining had likely faded; we generated recut H&E‐stained slides from the paraffin blocks. During the review process, slides with insufficient tumour cells or poor stain quality for AI learning were excluded, and one or a maximum of three histopathology slides were selected for each of the 338 patients. Without clinical information, whole slide images (WSIs) of 338 patients were used for the feature extractor model. Among the 338 patients, 102 whose clinical information was missing and 20 for whom the final response evaluation was not available were excluded (Figure 1). The 251 WSIs from 216 patients were divided using consecutive split validation into training and validation sets comprising 80% (200 patients) and a test set consisting of the remaining 51 patients. The review was conducted by two pathologists (MGN and YDC), and the selection of slides for this study was based on their consensus. The slides were scanned using a Leica‐Aperio GT450 Scanner with a ×40 objective (0.25 μm per pixel). Therefore, this study included 216 patients with 251 H&E‐stained WSIs and clinical information, including cancer recurrence and survival rates. The protocol for this retrospective study was approved by the Ethics Committee of the Institutional Review Board of Chonnam National University Hwasun Hospital in accordance with the Declaration of Helsinki (CNUHH‐2023‐225).

### Assessment of treatment response and survival

The treatment response was assessed using ^18^F‐PET/CT according to the Lugano response criteria for non‐Hodgkin lymphoma [22]. Interim PET/CT scans were obtained after 3–4 cycles of R‐CHOP, and end‐of‐treatment PET/CT was performed more than a month after completing immunochemotherapy. Interim and end‐of‐treatment PET/CT scans were compared with baseline PET/CT scans and evaluated according to visual assessment using Deauville criteria on a five‐point scale (DS): 1, no uptake; 2, uptake ≤mediastinum; 3, uptake >mediastinum but ≤liver; 4, uptake moderately increased compared with the liver uptake at any site; 5, markedly increased uptake compared with the liver at any site and new sites and/or new sites of disease. DS 1–3 were classified as complete response (CR) during the final response assessment. Progression‐free survival was defined as the time from diagnosis to disease progression or death from any cause, and overall survival (OS) was defined as the time from diagnosis to death from any cause. Non‐responders were defined as patients who did not achieve CR at the final response assessment.

### Self‐supervised learning for patch feature extraction

Self‐supervised learning techniques utilise intrinsic features of unlabelled data to obtain robust feature representation [23, 24, 25]. Notably, contrastive learning has emerged as a powerful method for analysing pathological images, enabling the extraction of high‐quality features from small patches. Specifically, the DINO method demonstrates exceptional ability to extract detailed features relevant to various cancer types, including cell morphology, tissue types, and histopathological structures [26, 27, 28]. In our approach, we extracted non‐overlapping 448 × 448 pixel patches from WSIs at ×40 magnification, subsequently downscaled to 224 × 224 pixels using Lanczos filtering. We then filtered out artifacts, such as non‐tissue background and extraneous noise, by assessing the pixel brightness. Additionally, we excluded areas where a depth‐first search identified that the contiguous region spanned 25 or fewer tiles.

Using the DINO model built on contrastive learning, we extracted the features from these patches. Our backbone model employed ViT‐S/8 [29], resulting in feature dimensions of 384. Despite the abundance of publicly available contrastive learning model weights, their application to DLBCL remains challenging. This is primarily because databases providing publicly available histopathological images rarely offer data on DLBCL subtypes. For instance, in The Cancer Genome Atlas (TCGA) database [30], which provides over 20,000 diagnostic slides, only 40 slides are available for the DLBCL subtype. As a result, we retrained the DINO model on patches from our DLBCL slides using the pre‐trained model developed by Kang *et al* [27]. Due to memory and GPU constraints, we set the batch size to 512 and used a low initial learning rate of 1.5e−6. We kept the other hyperparameters consistent with those implemented in the DINO study throughout our experiments. Finally, we visualised DINO's first component of feature representation using Uniform Manifold Approximation and Projection (UMAP) to preliminarily ascertain the quality of the features [31].

### MIL for slide‐level prediction

Recent algorithms based on MIL aim to predict the label of a ‘bag’ composed of instances, making them suitable methods for the classification of histopathology images. We utilised a dual‐stream multiple instance learning network with attention‐based pooling to consolidate information across all patches [32]. We utilised the attention score from attention‐based pooling as an indicator of patch importance, normalised these values with min–max scaling, and visualised them as a heatmap.

Gigapixel‐based histopathological images contain tens of thousands of patches. Given the relatively small size of our dataset, comprising 251 WSIs, overfitting is a significant concern in the model training process. To address this issue, we first implemented a multi‐modal model that integrates histopathology images with clinical data, anticipating that the predictive power of clinical data would stabilise training and mitigate overfitting on the training set. Consequently, 54 features extracted from clinical data using unsupervised learning with TabNet were combined with 384 features obtained after attention‐based pooling in the MIL model [33]. This composite feature set was then passed through the final linear layer for output prediction. Subsequently, a model solely based on pathology images was trained using knowledge distillation techniques derived from the multi‐modal model. The 384 features from the MIL model of the pathology image model were guided by the multi‐modal model's pathology image feature representation, using cosine similarity as a loss. For predicting slide‐level responses, we employed categorical cross‐entropy loss. The models were trained for 300 epochs with an initial learning rate of 0.0001, which was decreased by half if the validation loss did not decrease for 10 consecutive epochs. The model demonstrating the lowest loss on the internal validation set was selected.

### External validation dataset

TCGA stands as a pivotal public database, boasting a diverse array of clinical information, genomics, and image data. The TCGA pathology images used for external evaluation of the learned model were reviewed by two pathologists (MGN and YDC). In this investigation, we focused on histopathological image data, specifically FFPE slides used for patient diagnosis. Additionally, we integrated OS data into our study parameters. All slides were uniformly processed to yield patches with dimensions of 224 × 224 at a magnification of ×20. Using a model trained with the DINO architecture, we executed feature extraction. The TCGA dataset incorporates data from 40 patients, including follow‐up data, vital status records, age, sex, and clinical stage. Among the 48 patients with DLBCL, 36 received R‐CHOP treatment and 4 received other treatments. Based on the median values derived from our pathological model, we stratified individuals into two distinct categories. OS was subsequently assessed using Kaplan–Meier plots and log‐rank tests. In parallel, the Cox proportional hazard model was employed to juxtapose the clinical variables against pathology‐driven predictions. Of the 40 subjects, seven had documented mortality incidents, and the duration of follow‐up spanned up to 17.59 years.

### Statistical analysis

The model performance was assessed using receiver operating characteristic (ROC) curves and the associated area under the ROC curve (AUROC), with inter‐model AUROC differences determined using DeLong's test. Spearman's rank correlation was used to analyse the relationship between the predictive scores from the model and clinical variables. Binary outcomes were statistically evaluated using the Wilcoxon rank‐sum test (Mann–Whitney *U* test). Survival outcomes were visualised using the Kaplan–Meier method, with patients stratified into two groups based on predicted value medians for survival analyses, and the significance of survival differences was assessed using the log‐rank test. The Cox proportional hazard model was used to integrate clinical variables for survival analyses. All analyses were conducted using R software version 4.1.3. *p* values less than 0.05 were considered significant.

## Results

### Patients and dataset

The median age of the patients in the Chonnam National University Hospital dataset used for model training and internal validation was 66 years (range 20–87), and 95 patients (44.0%) were male (Table 1). One hundred and fifteen patients (53.2%) were in the Ann Arbor stage III–IV and 38 (17.6%) were classified as having a high‐risk IPI. Regarding treatment, 26 patients with limited stage disease received 3–4 cycles of R‐CHOP with or without IFRT, 141 received 6 cycles of R‐CHOP, and 45 received 8 cycles of R‐CHOP. Consolidation IFRT was done in 12 patients. After treatment, 186 patients (86.1%) were assessed as having CR, 9 (4.2%) had partial response, 2 (0.9%) had stable disease, and 19 (8.8%) had progressive disease. The demographic and clinical characteristics of the patients are listed in Table 1. The median age was higher in the non‐responder group, and the number of patients with elevated LDH, >2 extranodal involvement, and bulky masses was higher in the non‐responder group. As for the disease stage and IPI risk groups, more patients were assigned to the advanced stage and higher risk IPI in the non‐responder group.

**Table 1:** Demographics and clinical features of responders versus non‐responders

Variable	Total (*N* = 216)	Responder (*n* = 186)	Non‐responder (*n* = 30)	*p* value
Age, median (range)	66 (20–87)	65 (20–83)	71 (39–87)	0.004
Male (%)	95 (44.0)	81 (43.5)	14 (46.7)	0.843
Elevated LDH (%)	131 (60.6)	105 (56.5)	26 (86.7)	0.002
PS ≥2 (%)	28 (13.0)	22 (11.8)	6 (20.0)	0.240
Beta‐2 microglobulin	2,430.6 (348.0–17,871.0)	2,269.0 (348.0–17,871.0)	3,459.8 (1,957.0–9,007.0)	<0.001
B‐symptoms (+)	51 (23.6)	43 (23.1)	8 (26.7)	0.817
Extranodal involvement ≥2 (%)	48 (22.2)	35 (18.8)	13 (43.3)	0.005
Ann Arbor stage (%)				0.074
I	37 (17.1)	33 (17.7)	4 (13.3)	
II	64 (29.6)	60 (32.3)	4 (13.3)	
III	56 (25.9)	47 (25.3)	9 (30.0)	
IV	59 (27.3)	46 (24.7)	13 (43.3)	
BM involvement (%)	21 (9.7)	17 (9.1)	4 (13.3)	0.505
IPI (%)				0.001
Low	73 (33.8)	71 (38.2)	2 (6.7)	
Low–intermediate	53 (24.5)	47 (25.3)	6 (20.0)	
High–intermediate	52 (24.1)	41 (22.0)	11 (36.7)	
High	38 (17.6)	27 (14.5)	11 (36.7)	
Bulky mass (%)	13 (6.0)	8 (4.3)	5 (16.7)	0.021

BM, bone marrow; PS, performance status.

### Development of slide‐level drug response prediction model

**Figure 2:**
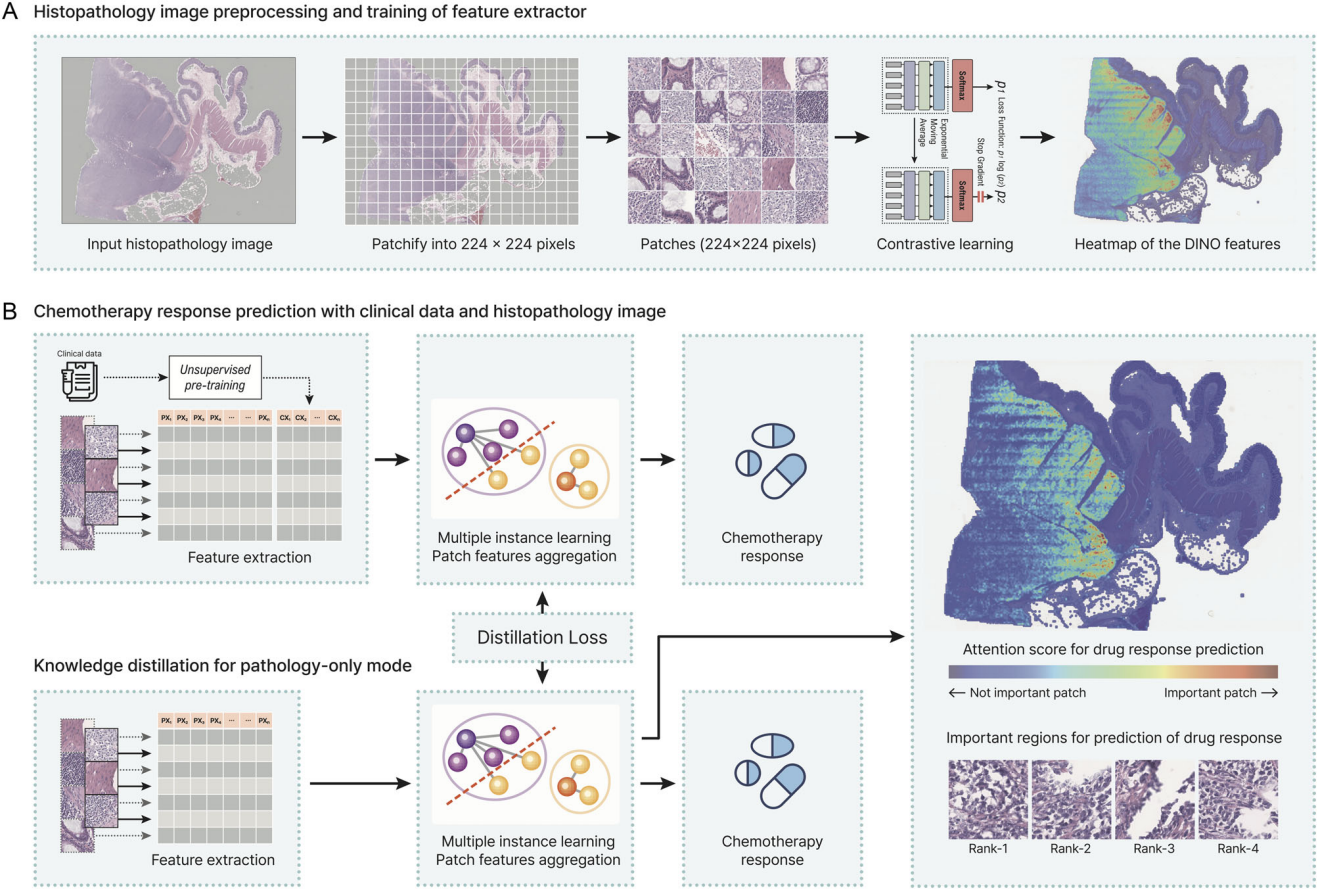
Schematic representation of the workflow for predicting CR to chemotherapy using histopathology images. (A) Histopathology image processing from patch generation to feature representation through contrastive learning. (B) Development of models to predict drug response using combined histopathology images and clinical data or using histopathology images alone. The histopathology‐only model was trained via knowledge distillation from the multi‐modal model, with an accompanying heatmap that underscores regions influencing the response through the attention mechanism.

A workflow scheme for model development to predict drug responses from histopathological images is shown in Figure 2. For visual insights into our feature extraction methodology, the DINO‐derived features were dimensionally reduced using UMAP (supplementary material, Figure S1). Through heatmaps of each UMAP component, a distinct demarcation between normal and cancerous tissues was evident. Additionally, within the cancerous regions, variations based on the unique texture of cancer cells were discernible. Although most cancer regions in DLBCL exhibited similar patterns, features derived from the DINO approach effectively distinguished the distinct textures of each cancer patch. In addition, our model, which leverages attention mechanisms, can pinpoint patches that have a substantial impact on the prediction of the presence or absence of a drug response.

### Performance evaluation of the prediction model

**Figure 3:**
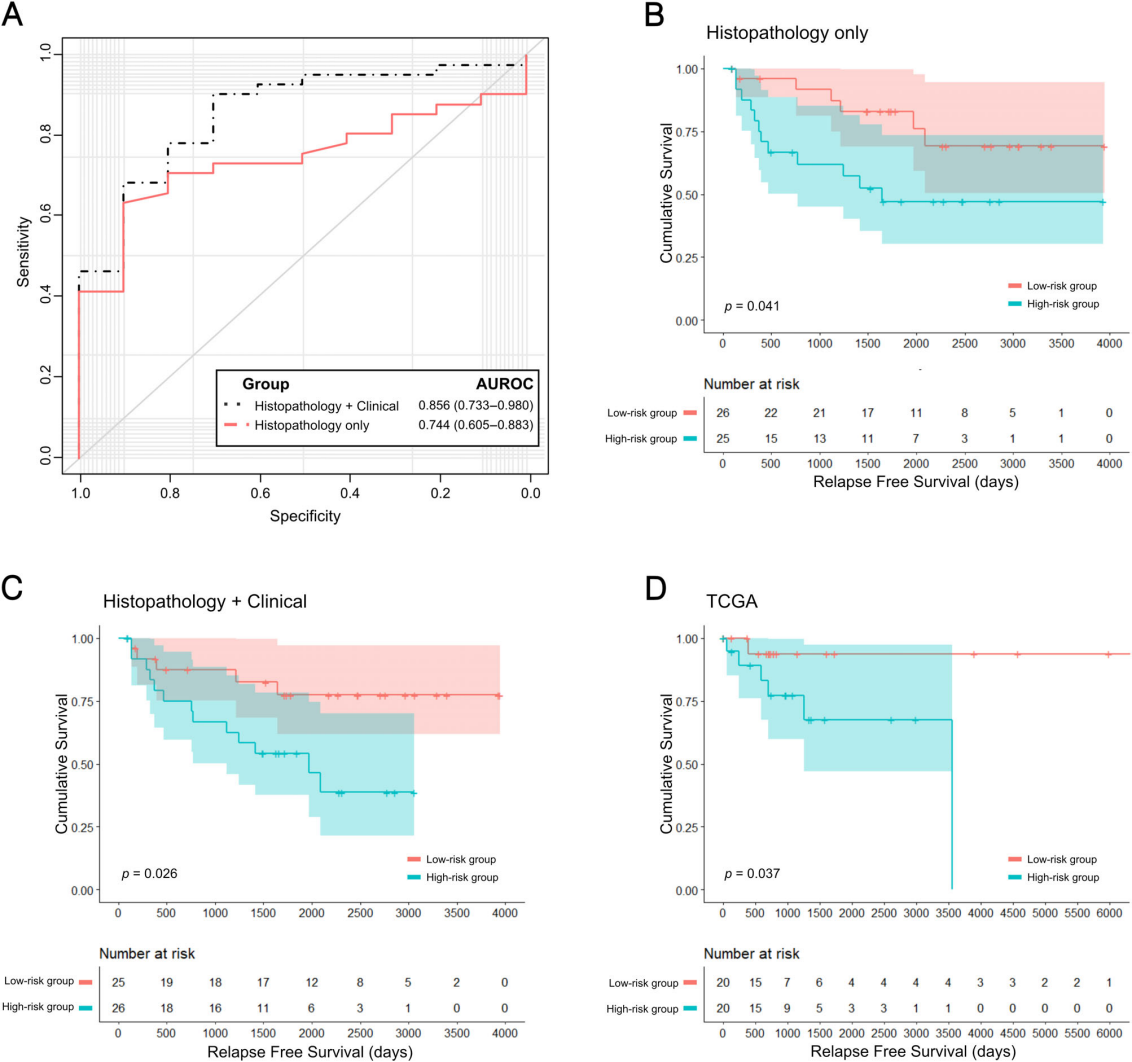
Performance of the model for drug response prediction and survival analysis. (A) ROC curves for drug response prediction by the histopathology‐based AI model and the multimodal AI model. (B) RFS analysis according to predictions from the histopathology‐based AI model. (C) RFS analysis conducted by the multi‐modal model. (D) External validation of RFS using TCGA dataset.

The model trained solely on pathology yielded an AUROC value of 0.744 (95% CI: 0.605–0.883), as depicted in Figure 3A. This model's sensitivity, specificity, positive predictive value (PPV), and negative predictive value (NPV) based on its Youden's index are 63.4%, 90.0%, 96.3%, and 37.5%, respectively. Additionally, the area under the precision‐recall curve (AUPRC) for this model is 0.935. The multi‐modal prediction model achieved an AUROC value of 0.856 (95% CI: 0.733–0.980). The sensitivity, specificity, PPV, and NPV for this model, based on its Youden's index, were 90.2%, 70.0%, 92.5%, and 63.6%, respectively. And the AUPRC for this model is 0.961.

### Survival analysis using AI predictions

The Kaplan–Meier plot (Figure 3B) demonstrates relapse‐free survival (RFS) outcomes predicted by an AI model trained solely on histopathology images. Using the log‐rank test for survival analysis, we observed a significant difference in survival between patient groups divided by the median AI prediction value (*p* = 0.041). The multimodal model, which integrates clinical variables, also significantly distinguished RFS outcomes, as indicated by a *p* value of 0.026 (Figure 3C).

### External validation through survival analysis in TCGA

For external validation using the TCGA dataset, we observed a statistically significant survival difference with a *p* value of 0.037 (Figure 3D). To assess the prognostic significance of pathology‐based prediction, even when combined with clinical variables, we conducted a Cox proportional hazards analysis incorporating age, sex, and clinical stage. Although the pathology‐based prediction did not achieve statistical significance, it displayed the lowest *p* value among all the clinical factors, accompanied by a positive coefficient (supplementary material, Table S1). Of the seven recorded deaths, only one individual belonged to the group anticipated to respond well to drug treatment.

### Drug response prediction and associated variables

**Figure 4:**
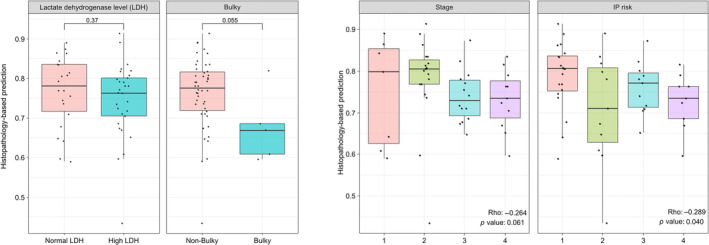
Associations between histopathology‐based model predictions and clinical variables.

We analysed clinical variables in relation to the predictions of the model that exclusively utilised histopathology (Figure 4). The association between LDH level and histopathology‐based predictions was not significant (*p* = 0.37). The *p* value for bulky disease was borderline at 0.055. For Ann Arbor stage, Spearman correlation analysis showed a *ρ* value of −0.264, indicating a negative correlation with a *p* value of 0.061. Similarly, the IPI risk exhibited a *ρ* value of −0.289, suggesting a negative correlation with a significant *p* value of 0.040.

### Histological features related to drug response

**Figure 5:**
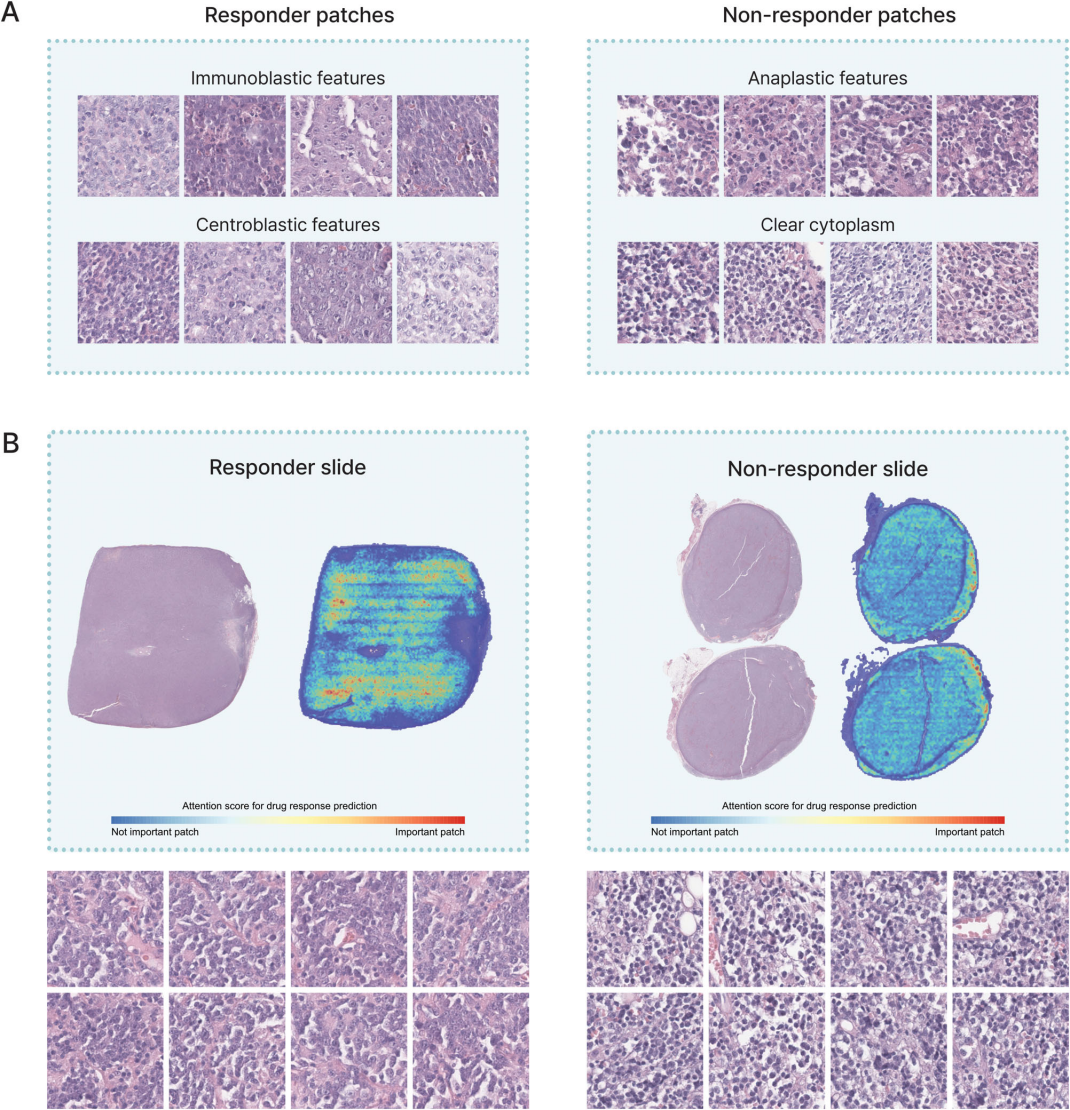
Visual representation of the model predicting drug response. (A) Classification of principal patches based on histological characteristics in relation to drug response. (B) Comprehensive heatmap of the slide according to drug responses, and patches with the highest attention score.

Patches highlighted from the slides that were predicted by the deep learning model to have a CR were categorised based on their histological characteristics. Our multiple instance model allowed us to retrieve the most predictive patches from thousands of processed patches. We investigated key histological determinants by extracting the 4020 most predictive patches (3040 for responders and 980 for non‐responders) from 216 WSIs using a prediction model and reviewing them by expert pathologists (MGN and YDC). Immunoblastic features and centroblastic features were observed in patches that best predicted responders, and anaplastic features and a clear cytoplasm were observed in patches that best predicted non‐responders (Figure 5A). Overall, these results demonstrate that our multiple instance model can detect histological patterns associated with chemotherapy responsiveness and survival in patients with DLBCL. We further created a heatmap distribution of instances for each WSI and examined the overall distribution of the predicted risk for each patch (Figure 5B). We then investigated whether the histological characteristics of patches with a high contribution to responders or non‐responders were distributed within each WSI. In the responder WSI, the anaplastic features or clear cytoplasmic features were distributed as a high signal in the heatmap within the WSI, and in the non‐responder WSI, the immunoblastic feature or centroblastic feature was distributed as a high signal in the heatmap within the WSI.

## Discussion

The objective of this research was to create a deep learning model for predicting immunochemotherapy responses in DLBCL based on H&E histopathology images. Based on knowledge distillation, our model could avoid overfitting to gigapixel histopathology images to predict immunochemotherapy response. To the best of our knowledge, this study is the first attempt to predict drug responses in DLBCL using histopathology.

This study addresses two significant challenges compared with previous research on digital pathology image analysis. First, while AI research has focused primarily on automating tasks typically performed by humans, such as tumour subtype classification and identification of metastases, this study addresses the highly demanding task of predicting immunochemotherapy response [32, 34], which is not a task that can typically be performed accurately by human experts. We validated the performance of our model using both internal and external datasets, supported by a survival analysis. Second, WSIs of the breast, lung, prostate, and other tumour types typically studied contain structural/tissue architectural features within the slides, whereas DLBCL lacks substantial structural/architectural characteristics [18, 19]. Given the similarity in structural patterns across most DLBCL patches, a feature extractor with exceptional performance is required. Previous studies have employed features extracted from CNN models trained on ImageNet data, such as ResNet and EfficientNet. However, these models may not yield favourable results for DLBCL patches because of their highly similar structural patterns. To achieve a robust performance and mitigate overfitting, we implemented a model that utilised contrastive learning and knowledge distillation.

Several proven clinical factors for predicting prognosis in DLBCL exist, such as the IPI, Ann Arbor stage, performance status, age, bulky disease, and serum LDH levels. In the present study, the prediction model based on histopathology showed a correlation with Ann Arbor stage, IPI risk, and bulky disease, although there was borderline significance in bulky disease and no significant correlation with serum LDH levels. These classical prognostic factors were developed and validated before the addition of rituximab to anthracycline‐based chemotherapy. Because rituximab considerably improves the treatment response and survival of patients with DLBCL, the influence of clinical factors such as LDH and bulky disease on predicting treatment response is somewhat reduced. This might explain the weak correlation between these clinical factors and the prediction model in this study, because all included patients were treated with rituximab‐containing immunochemotherapy. However, the anticipated response group according to the histopathological prediction model of this study showed significantly better RFS, which suggests that our study model is effective in predicting treatment response to immunochemotherapy and provides an independent prognostic implication for patients with DLBCL in the rituximab era.

According to the previous World Health Organization (WHO) (2008) tumour classification, morphological variants of DLBCL include centroblastic, immunoblastic, and anaplastic subtypes, of which the centroblastic subtype is the most common and is known to have a better prognosis and higher OS [35, 36, 37, 38, 39]. However, several biologically and clinically heterogeneous cases remain for which there are no clear and acceptable criteria for sub‐classification, and these cases are now collectively referred to as DLBCL, not otherwise specified [40, 41, 42]. In this study, centroblastic and immunoblast‐type characteristics were observed in patches from the WSI of the responder group, whereas the anaplastic subtype and clear cytoplasm were observed within the WSI of the non‐responder group. This is consistent with what was proposed in the findings of a previous WHO tumour classification. Morphological classification by pathologists is subject to inter‐ and intra‐observer differences in interpretation, which compromises diagnostic objectivity and reproducibility. AI‐based deep learning using digital pathology images can overcome these shortcomings and increase objectivity and reproducibility. We externally validated our results using TCGA data; however, the performance of our model could be further improved and validated using additional multi‐centre datasets.

In this study, we demonstrated the effectiveness of a multiple instance learning method using clinical information and pathology image data. This multi‐modal integration improves both the classification performance and computational efficiency. However, this study has several limitations. First, we initially targeted 729 patients with DLBCL and ultimately used 216 WSIs. In addition, the learning process was performed using DINO with WSI without specific annotations to distinguish between neoplastic and non‐neoplastic areas within a WSI. Previous studies have shown that incorporating appropriate guidance biases can significantly improve model performance [16]. In particular, among the 216 patients used as study subjects, the organ in which DLBCL occurred was not just the lymph nodes. Although the shape and histological characteristics of tumour cells may have been similar for DLBCL occurring in various organs of the body, the background non‐neoplastic tissues may have been very different; therefore, these factors may have influenced the model's training and subsequent performance. Given the requirement for expert haematopathologists willing to perform manual annotations, as well as the labour‐intensive and time‐consuming nature of manual delineation of tumour regions, we did not incorporate a tumour segmentation step in the current study. However, this might be a reasonable addition to future versions of the model. Despite the lack of a tumour region segmentation step, our model generalised well to the external (TCGA) dataset.

DLBCL is a heterogeneous disease, not only in terms of clinicopathology, but also in terms of molecular and genetic characteristics. This study used only clinical and digital pathology data, including data from H&E‐stained slides. However, DLBCL is not actually diagnosed with H&E staining alone; it requires additional molecular pathological tests, including immunohistochemical staining, for diagnosis. It is expected that if we perform multi‐modal learning on various types of data, including immunohistochemically stained digital slides, other test results, and molecular genetic data, we will be able to create a prediction model with better performance.

## Author contributions statement

M‐GN and D‐HY developed the study concept and design. JHL conducted the data analysis and carried out the experiments for model development. G‐YS and KMM were responsible for preparing the manuscript. JS and JL contributed by reviewing the manuscript and preparing responses to reviewer comments. S‐RK provided the material support. Y‐DC and M‐GN performed histopathological review. All authors read, edited and approved the final manuscript.

## Supporting information


**Figure S1.** UMAP result based on DINO patch feature representation
**Table S1.** Cox proportional hazards analysis of pathology prediction with clinical variables in TCGA

## Data Availability

The datasets generated and/or analysed during the current study are available from the corresponding author upon reasonable request.
