# Supplementary material for: Prediction of immunochemotherapy response for diffuse large B‐cell lymphoma using artificial intelligence digital pathology
Source: J Pathol Clin Res. 2024 Apr 8;10(3):e12370. doi: 10.1002/2056-4538.12370 (PMC10999948; doi:10.1002/2056-4538.12370)
Supplement: Supplementary file 1 — Figure S1. UMAP result based on DINO patch feature representation Table S1. Cox proportional hazards analysis of pathology prediction with clinical variables in TCGA [file CJP2-10-e12370-s001.pdf]

Prediction of immunochemotherapy response for diffuse large B-cell lymphoma using artificial intelligence digital pathology

JH Lee, G-Y Song *et al.*, *J Pathol Clin Res*, https://doi.org/10.1002/2056-4538.12370

Supplementary Figure S1

Supplementary Table S1

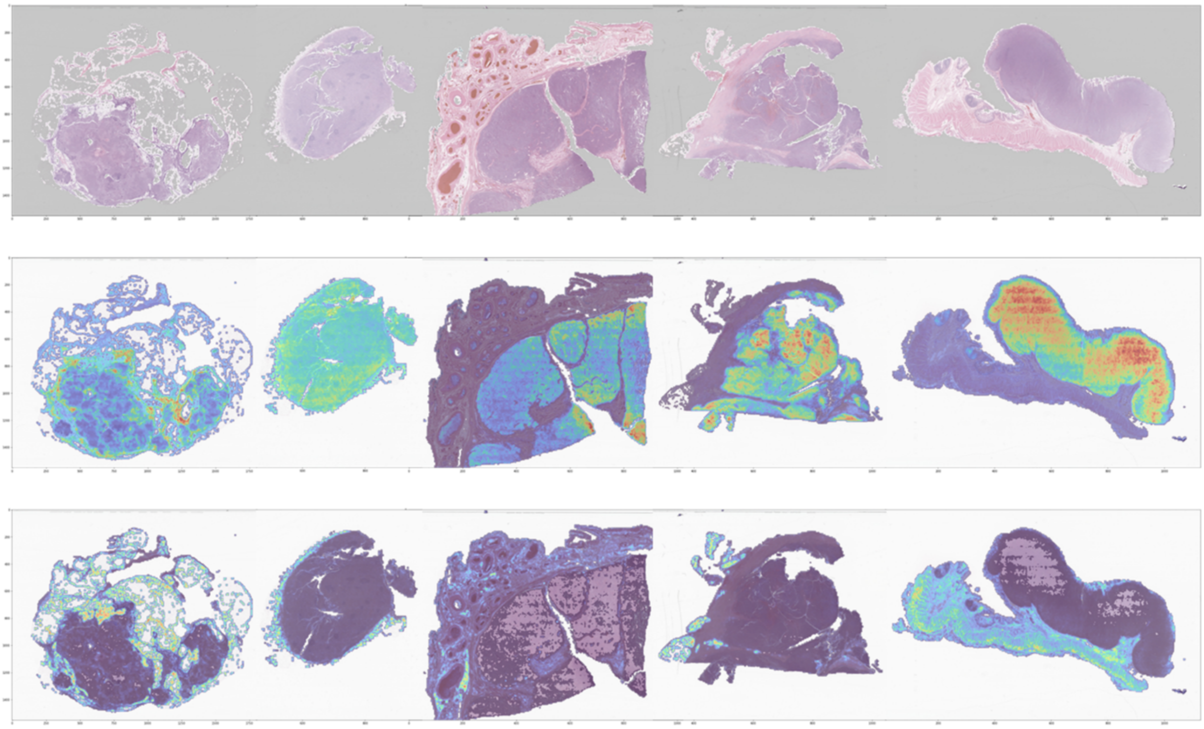

Figure S1. UMAP result based on DINO patch feature representation.

Table S1. Cox proportional hazards analysis of pathology prediction with clinical variables in TCGA

**Variable name**	**n**	**coef**	**Hazard ratio**	**se(coef)**	**z**	***p*.value**
Pathology-prediction		-0.919	0.399	0.567	-1.620	0.105
Age		-0.056	0.946	0.036	-1.575	0.115
Gender (reference: male)	22					
Female	18	-0.941	0.390	1.224	-0.769	0.442
Stage (reference: Stage I)	8					
Stage II	14	0.431	1.539	1.276	0.338	0.735
Stage III	4	1.311	3.709	2.024	0.648	0.517
Stage IV	8	0.650	1.916	1.338	0.486	0.627
